# Mast cells and histamine are triggering the NF-κB-mediated reactions of adult and aged perilymphatic mesenteric tissues to acute inflammation

**DOI:** 10.18632/aging.101113

**Published:** 2016-11-21

**Authors:** Irina Tsoy Nizamutdinova, Giuseppina F. Dusio, Olga Yu. Gasheva, Hunter Skoog, Richard Tobin, Chander Peddaboina, Cynthia J. Meininger, David C. Zawieja, M. Karen Newell-Rogers, Anatoliy A. Gashev

**Affiliations:** ^1^ Department of Medical Physiology, College of Medicine, Texas A&M University Health Science Center, Temple, TX 76504, USA; ^2^ Department of Surgery, Baylor Scott and White Health, Texas A&M University Health Science Center, Temple, TX 76504, USA

**Keywords:** aging, mast cell, lymphatic vessel, histamine, NF-κB signaling

## Abstract

This study aimed to establish mechanistic links between the aging-associated changes in the functional status of mast cells and the altered responses of mesenteric tissue and mesenteric lymphatic vessels (MLVs) to acute inflammation. We used an *in vivo* model of acute peritoneal inflammation induced by lipopolysaccharide treatment of adult (9-month) and aged (24-month) F-344 rats. We analyzed contractility of isolated MLVs, mast cell activation, activation of nuclear factor-κB (NF-κB) without and with stabilization of mast cells by cromolyn or blockade of all types of histamine receptors and production of 27 major pro-inflammatory cytokines in adult and aged perilymphatic mesenteric tissues and blood. We found that the reactivity of aged contracting lymphatic vessels to LPS-induced acute inflammation was abolished and that activated mast cells trigger NF-κB signaling in the mesentery through release of histamine. The aging-associated basal activation of mesenteric mast cells limits acute inflammatory NF-κB activation in aged mesentery. We conclude that proper functioning of the mast cell/histamine/NF-κB axis is necessary for reactions of the lymphatic vessels to acute inflammatory stimuli as well as for interaction and trafficking of immune cells near and within the collecting lymphatics.

## INTRODUCTION

Aging is associated with a greater susceptibility to infections due to the functional decline in the immune system, also known as immunosenescence [1]. While networks of lymphatic vessels provide the sole route for immune cells to travel from peripheral tissues towards lymph nodes [2–4], knowledge of how aging affects these networks remains rudimentary. In particular, mesenteric lymphatic vessels (MLVs), being located at the border between the potentially biologically aggressive environment of the gut lumen and inner compartments of the abdomen, may be influenced by pathogens delivered from the gut. However, until recently, these lymphatic vessels have not been studied in depth with regard to the aging-associated alterations of their principal, from birth to death existing, role – the maintenance (via intrinsic phasic contractility) of effective movement of lymph fluid (and immune cells within it) towards the lymph nodes. Moreover, the mechanisms underlying the interactions between MLVs and immune cells in the surrounding mesenteric tissue microenvironment remain, to a large degree, unknown. The aging-induced specificity of such interactions during acute gut and peritoneal inflammation has not been studied. This limits progress in development of better therapies for treating the elderly population affected by acute inflammation due to the lack of consideration of aging-related changes in the maintenance of lymph flow, a necessary component of the body's defense against infection.

We previously demonstrated aging-associated weakening of lymphatic contractility in MLVs [5, 6]. Therefore, the reduced mesenteric lymph flow in the elderly, by default, will predictably impair the acute inflammatory response of aged MLVs, by altering immune cell trafficking inside lymphatic vessels. Further studies revealed that inhibitory effects of disturbed nitric oxide (NO) production in aged lymphatic vessels [6, 7] coexisted with the abnormal functional status of certain [at that time undefined] cellular elements in the mesenteric perilymphatic tissue microenvironment. In subsequent studies, we discovered increased aging-associated basal activation of mast cells located in tissues adjacent to MLVs [8]. Additionally, we found that the aging-associated chronic activation of mast cells limits the recruitment towards MLVs and subsequent activation of MHC II+ cells as well as the recruitment of eosinophils towards MLVs during acute inflammatory stimulation [9]. We proposed [9] that this occurs because of limitations in the acute release by aged mast cell of mediators necessary for modulation of the immune response [10–12]. Based on these findings, we hypothesized that during the initial phase of acute inflammation in aged mesenteric perilymphatic tissues, this pre-existing aging-associated chronic partial degranulation of peri-lymphatic mast cells causes abnormalities of lymphatic functions and the immune response in the elderly population, thereby diminishing the effectiveness of the aged body's response to acute gut and peritoneal inflammation.

Considering the underlying molecular mechanisms involved in these processes, we focused our attention on the well-established fact that activated nuclear factor-κB (NF-κB) is a major factor in the pathogenesis of aging-induced, low-grade chronic inflammation [13–20]. At the same time, NF-κB is a key component of the body responses to acute inflammation via its main regulatory role in the production of numerous cytokines [21–26]. Upon acute and chronic inflammation-related activation, mast cells can release numerous vasoactive and inflammatory mediators that influence the surrounding tissues, including the MLVs and populations of various immune cells located close to MLVs [10, 11, 27–30]. Some reports indicate that mast cell-derived factors may trigger the NF-κB pro-inflammatory cascade in the surrounding tissues [31, 32]. Histamine, for example, is known for its ability to activate NF-κB signaling and NF-κB-dependent release of TNF-α and IL-6 in synovial tissue and microglia [33, 34]. However, knowledge of the potential linkage between activated mast cells, histamine and NF-κB activation upon development of acute or chronic inflammation in the mesentery does not exist in the literature. Importantly, the effects of aging on these mechanisms are unknown and therefore have not been considered at all.

In this study, our aims were to obtain evidence to support our hypothesis that pre-existing aging-associated degranulation of perilymphatic mast cells induces abnormalities of lymphatic functions and the immune response in elderly individuals and to investigate the underlying molecular mechanisms. To achieve these goals we used an *in vivo* model of acute (24-hr) peritoneal inflammation induced by intra-peritoneal (IP) injection of lipopolysaccharide (LPS) in adult (9-mo old) and aged (24-mo old) rats as well as *in vitro* models with LPS treatment. We evaluated aging-associated changes in the contractile transport function of mesenteric lymphatic vessels and in the functional status of the adjacent mast cells before and after development of acute peritoneal inflammation. We also performed experiments to establish the mechanistic links between mast cell activation and the triggering of the NF-κB signaling in the mesenteric tissues of adult and aged animals. Finally, we evaluated the aging-induced changes on the body's responses to acute inflammation, in terms of specific cytokine production with reference their potential sources in the aged and inflamed mesentery.

## RESULTS

### Abolished reactivity of aged contracting lympha-tic vessels to LPS-induced acute inflammation

**Figure 1:**
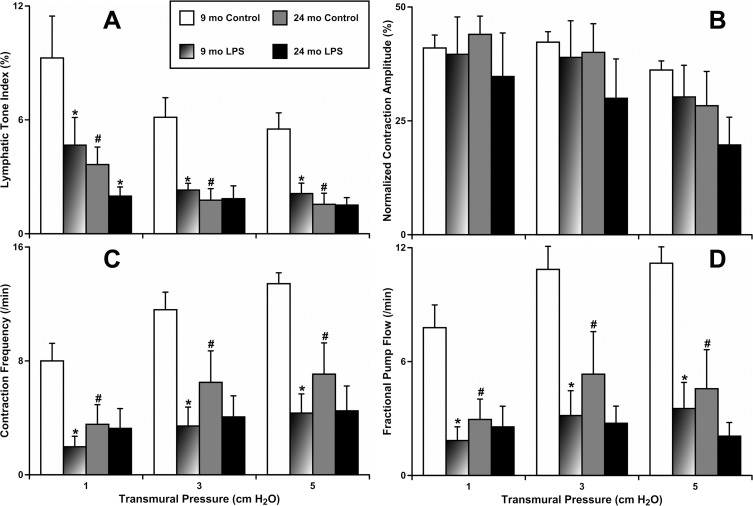
Effects of LPS-induced acute inflammation on parameters of contractility of adult (9 mo, n=6 for control and n=6 for LPS-treated groups) and aged (24 mo, n=6 for control and n=6 for LPS-treated groups) mesenteric lymphatic vessels (**A**) lymphatic tone index; (**B**) contraction amplitude; (**C**) contraction frequency; (**D**) fractional pump flow. * indicates significant differences (p < 0.05, one-way ANOVA) between control and LPS-treated lymphatic vessels within each age group at any value of transmural pressure. # indicates significant differences (p < 0.05, one-way ANOVA) between adult and aged lymphatic vessels in control group at any value of transmural pressure.

To evaluate whether aging influences the reactivity of contracting MLVs in response to 24 hours of LPS-induced inflammation, we incubated freshly isolated segments of mesentery containing MLVs obtained from animals of both ages with vehicle or LPS-containing solution. Subsequently, we isolated the MLVs from these segments of mesentery and characterized their contractile activity. Figure 1 presents findings obtained in these experiments. All parameters of contractile activity of MLVs, in both 9-mo and 24-mo rats under control conditions, matched those described for these age groups in the past under different experimental settings [5, 6, 35]. These findings validated our current approach of utilizing ex vivo tissue segments kept 24 hours under culture conditions with and without LPS administration. Effects of the 24 hours of LPS-induced inflammation on the contractile parameters of MLVs obtained from adult animals (9-mo) were similar to those obtained in MLVs isolated from younger (~3 mo) animals that had either undergone 24 hours of LPS treatment *in vitro* or 72 hours of LPS treatment *in vivo* [36]. Our findings from 9-mo animals demonstrated a 58% lowering of lymphatic tone (Fig. 1A); 71% decreased lymphatic phasic contraction frequency (Fig. 1C) and 72% decrease in lymphatic minute pumping (Fig. 1D) as a result of acute LPS-induced inflam-mation. At the same time, in aged MLVs, the acute inflammation did not induce changes in these parameters of lymphatic phasic contractility, demonstrating only slight trends toward additional (to aging-associated) inhibition (Fig. 1 B-D). The lymphatic tone was significantly reduced in aged MLVs only at the lower level of their filling (intraluminal pressure 1 cm H_2_O, Fig. 1A). Cumulatively, these data demonstrate that aged MLVs have abolished their reactivity to the LPS-induced acute peritoneal inflammation compared to MLVs from adults.

### Diminished activation of aged mast cells during LPS-induced acute inflammation

**Figure 2:**
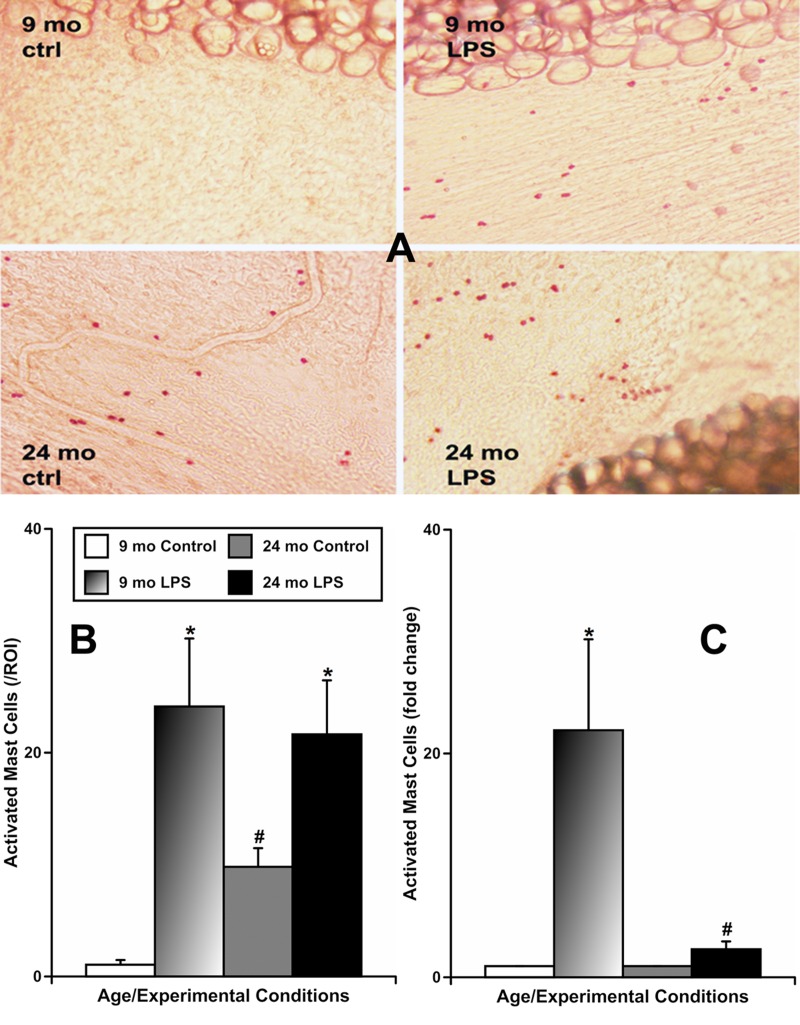
Effects of LPS-induced acute inflammation on activation of mast cells located in mesentery close to mesenteric lymphatic vessels in adult (9 mo, n=4 for control and n=4 for LPS-treated groups) and aged (24 mo, n=4 for control and n=4 for LPS-treated groups) rats (**A**) representative images of activated mast cells stained by Ruthenium Red in sham (ctrl) and LPS-treated mesenteric segments of both ages; (**B**) comparison of number of activated mast cells per region of interest (ROI) under various conditions; (**C**) similar data normalized to control conditions for that age group to demonstrate the fold change in number of mast cells activated by LPS. * indicates significant differences (p < 0.05, one-way ANOVA) between control and LPS-treated mesenteric segments within each age group. # indicates significant differences (p < 0.05, one-way ANOVA) between adult and aged mesenteric segments in control group or in LPS-treated group.

To evaluate whether aging influences the activation of mast cells located by MLVs in response to LPS-induced inflammation, we used two approaches. In one set of experiments we incubated freshly isolated segments of mesentery from animals of both ages containing MLVs overnight with vehicle or LPS-containing solution. Subsequently, we stained all segments with Ruthenium Red, which is able to enter and selectively stain only activated mast cells, as we previously described [8]. In this set of experiments (representative images shown in Fig. 2A), we found that mesenteric tissues from 9-mo animals had a low number of activated mast cells under resting conditions (1.0±0.4 cells/ROI [region of interest]), while LPS treatment markedly increased the number of activated mast cells (24.1±6.1 cells/ROI). At the same time, mesenteric tissues from 24-mo rats, under resting conditions, already had a large number of activated mast cells (9.8±1.7 cells/ROI). We found a further increase in the number of activated mast cells in 24-mo rat mesenteric segments in response to LPS treatment (21.6±4.8 cells/ROI), reaching a number similar to that of activated mast cells in 9-mo rat mesenteric segments treated with LPS. These findings are presented in Figure 2B. In adult mesenteric tissue segments, LPS induced a 22±8-fold increase in mast cell activation, while in aged mesenteric tissue segments we observed only a 2.5±0.7-fold increase (Figure 2C) presumably reflecting the consequences of the pre-existing increased basal activation of aged mesenteric mast cells. Cumulatively, these data demonstrate that the diminished reactivity of the mesenteric mast cells located close to MLVs in aged animals to acute inflammation is due to the limited number of mast cells available to be activated acutely in the aged mesentery.

**Figure 3:**
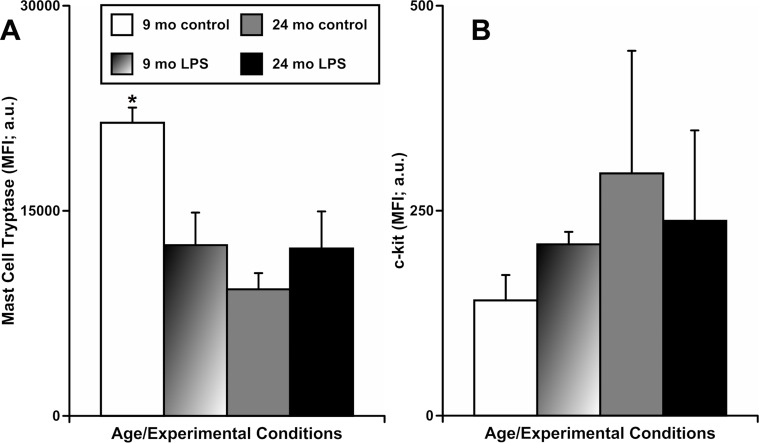
Effects of LPS-induced acute inflammation on the mean fluorescence intensity (MFI) of mast cell tryptase (A) and c-kit (B) signals in cells double positive for mast cell tryptase and c-kit that are present in mesentery close to mesenteric lymphatic vessels in adult (9 mo, n=3 for control and n=3 for LPS-treated groups) and aged (24 mo, n=3 for control and n=3 for LPS-treated groups) rats * indicates significant difference (p < 0.05, one-way ANOVA) between MFI of the mast cell tryptase signal in 9 mo control group vs other groups.

In another set of experiments, we evaluated the changes in the functional status of mast cells located by MLVs using flow cytometric analysis of mesenteric tissue segments of animals of both ages, before and after acute peritoneal inflammation. In these experiments, we determined the frequency of live mast cells based on cytometric gating strategies combined with staining of the mast cells with antibodies recognizing mast cell tryptase (MCT) and c-kit as confirmed markers of mast cells [8]. We did not find significant differences in the number of live MCT/c-kit-double positive cells between groups of specimens, when considering either age or presence of peritoneal inflammation induced by LPS treatment (data not shown). Since the loss of MCT expression is an indicator of their activation and degranulation [37–39], we also analyzed the mean fluorescence intensity (MFI) of the MCT (Figure 3A) and c-kit (Figure 3B) signals in these live mast cells. We found (Figure 3A) that the MFI of MCT was significantly higher in mast cells from the 9-mo control group - 21440±1111 arbitrary units [a.u.] than in mast cells from all other groups: 12490±2378 a.u. (9-mo LPS-treated), 9257±1186 a.u. (24-mo control) and 12254±2709 a.u. (24-mo LPS-treated). The c-kit MFI (Figure 3B) was not significantly different in mast cells from all groups, as expected. Cumulatively, these data additionally indicate that the abnormal reactivity of the mesenteric mast cells located near MLVs in aged animals to acute inflammation appears to be due to pre-existing basal activation of these mast cells (evidenced by depletion of intracellular MCT).

### Diminished activation of NF-κB in aged mesentery during LPS-induced acute inflammation

**Figure 4:**
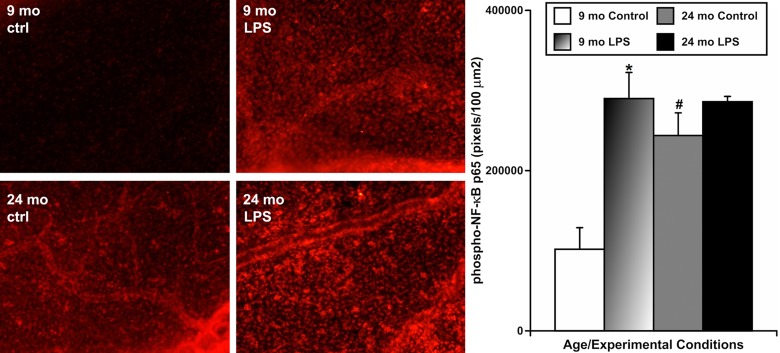
Effects of LPS-induced acute inflammation on activation of NF-κB signaling in mesentery close to mesenteric lymphatic vessels in adult (9 mo, n=4 for control and n=4 for LPS-treated groups) and aged (24 mo, n=4 for control and n=4 for LPS-treated groups) rats Left panels – representative images of mesentery labeled for phospho-NF-κB p65 (in red) in sham (ctrl) and LPS-treated (LPS) mesenteric segments of both ages. The graph on the right shows a comparison of pixel intensity of phospho-NF-κB p65 per 100 μm^2^ under the various conditions. * indicates significant difference (p < 0.05, one-way ANOVA) between control and LPS-treated mesenteric segments within each age group. ^#^ indicates significant difference (p < 0.05, one-way ANOVA) between adult and aged mesenteric segments under control conditions.

To evaluate whether aging influences the activation of NF-κB in mesenteric tissues near the MLVs under resting conditions and in response to the LPS-induced inflammation, we also used animals of both ages in which we induced acute peritoneal inflammation by a single IP injection of LPS, as described above. After 24 hours, we performed immunohistochemical labeling of phosphorylated NF-κB (p65) in segments of mesentery containing MLVs to evaluate the activation of this signaling pathway [40, 41]. In this set of experiments (representative images shown on Figure 4), we found that mesenteric tissues from 9-mo animals expressed the lowest level of NF-κB activation under resting conditions (mean pixel intensity 101846±26942 pixels/100 μm^2^) while LPS treatment increased the level of NF-κB activation in 9-mo mesenteric tissues dramatically (~2.8-fold, i.e. 289827±32631 pixels/100 μm^2^). At the same time, mesenteric tissues from 24-mo rats under resting conditions already had a significantly higher level of NF-κB activation than their adult counterparts (~2.4-fold, i.e. 243742±28328 pixels/100 μm^2^). Subsequently, we found only a slight, but non-significant, increase of NF-κB activation in aged 24-mo rat mesenteric segments after LPS treatment (285927±6600 pixels/100 μm^2^). This level was almost identical to the level of NF-κB activation in adult 9-mo rat mesenteric segments under the same treatment conditions. These findings are represented in the graph on Figure 4. Cumulatively, these data demonstrate that the pre-existing NF-κB activation in aged mesentery under resting conditions is remarkably high and there is no significant increase in NF-κB activation in the aged mesenteric tissues located close to MLVs in response to acute inflammation.

### NF-κB activation in mesenteric tissues is dependent on activation of mast cells and histamine in both adult and aged animals

**Figure 5:**
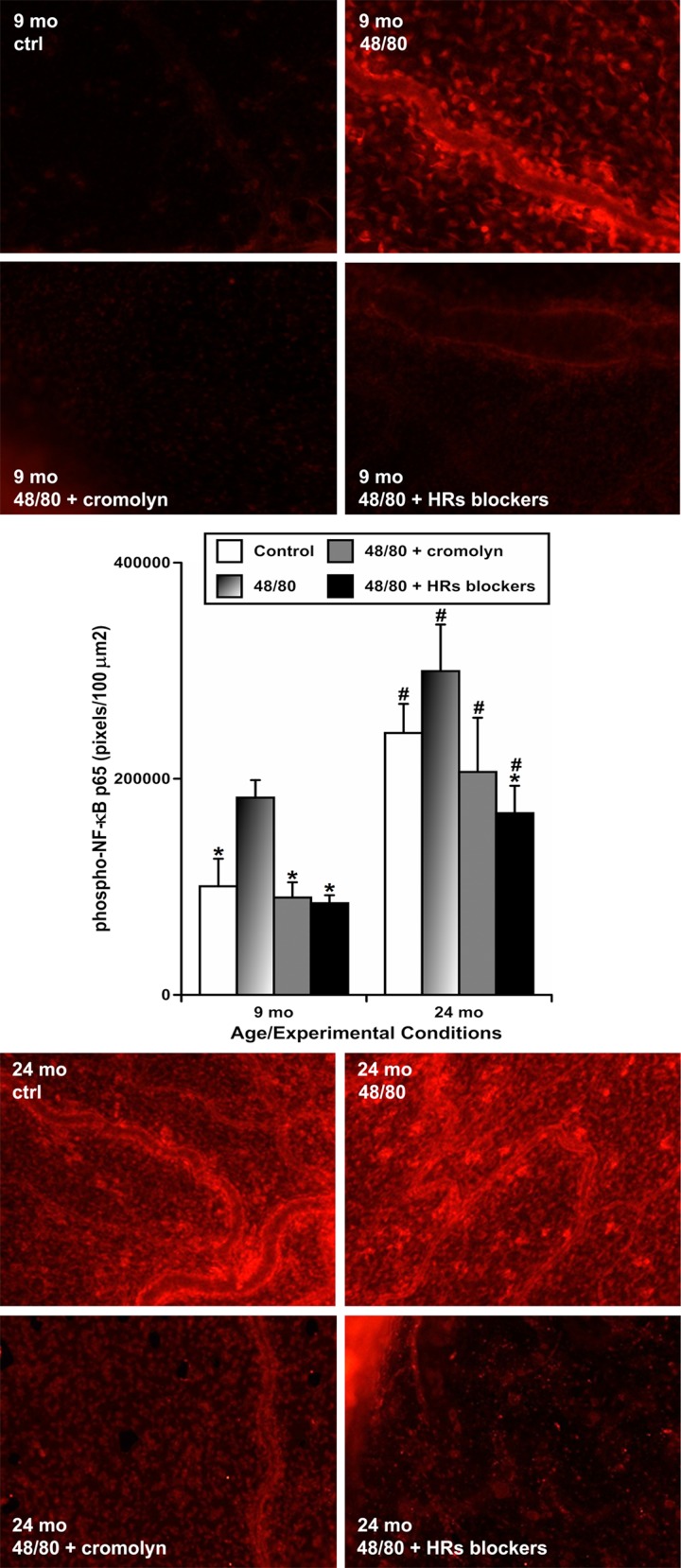
Effects of chemically-induced (compound 48/80) activation of mesenteric mast cells on activation of NF-κB in mesentery close to mesenteric lymphatic vessels in adult (9 mo, n=4 for control and n=4 for each treatment group) and aged (24 mo, n=4 for control and n=4 for each treatment group) rats without and with coinciding blockade of mast cell activation by cromolyn, or blockade of all types of HRs using a mixture of HR blockers Upper panels – representative images of adult (9-mo) mesenteric segments labeled for phospho-NF-κB p65 (in red) in sham (ctrl) conditions and following treatment with 48/80, 48/80 with cromolyn and 48/80 with HR blockers. Lower panels demonstrate images of aged (24-mo) mesenteric segments labeled for phospho-NF-κB p65 (in red) after the similar treatments. Graph in the middle shows a comparison of pixel intensity of phospho-NF-κB p65 per 100 μm^2^ under various conditions in the two age groups. * indicates significant difference (p < 0.05, one-way ANOVA) between 48/80-treated mesenteric segments and segments under all other treatments within each age group. ^#^ indicates significant difference (p < 0.05, one-way ANOVA) between adult and aged mesenteric segments in groups with similar treatments.

To evaluate whether NF-κB activation in the mesenteric tissues depends on mast cell activation and histamine release upon initiation of acute peritoneal inflammation, and if aging influences these relationships, we incubated freshly isolated segments of mesentery containing MLVs from animals of both ages and subjected them to the following 4 treatments: (1) sham control; (2) treatment with compound 48/80 to induce activation of mast cells; (3) pre-treatment with cromolyn sodium in order to block mast cell activation and to inhibit histamine secretion from mast cells, then treatment with compound 48/80 plus cromolyn; and (4) pre-treatment with a mixture of histamine receptor (HR) blockers in order to block all 4 types of HRs, then treatment with compound 48/80 plus HR blockers. Afterwards, we performed immunohistochemical labeling of phospho-rylated NF-κB (p65). In this set of experiments (representative images shown in Figure 5), we found that mesenteric tissues from 9-mo animals exhibit the lowest level of NF-κB activation under resting conditions (mean pixel intensity 100445±25541 pixels/100 μm^2^) while compound 48/80 treatment significantly increased the level of NF-κB activation in 9-mo mesenteric tissues (~1.8-fold increase, i.e. 182476±16250 pixels/100 μm^2^). Both blockade (by cromolyn) of mast cell activation (90035±14143 pixels/100 μm^2^) and blockade of HRs (84764±7424 pixels/100 μm^2^) kept levels of NF-κB activation similar to those of the control 9-mo rat mesenteric segments. The mesenteric tissues from 24-mo rats, under resting conditions, already had a significantly higher level of NF-κB activation than their adult counterparts (~2.4-fold increase, i.e. 242342±26927 pixels/100 μm^2^). Consequently, the treatment with compound 48/80 in aged 24-mo rat mesenteric segments produced only a slight but non-significant increase in NF-κB activation (299623±43087 pixels/100 μm^2^). In aged segments, both cromolyn-induced blockade of mast cell activation (206275±50225 pixels/100 μm^2^) and blockade of HRs (168067±25458 pixels/100 μm^2^) were able to prevent any compound 48/80-induced acute NF-κB activation. In addition, while MC stabilization led to a non-significant trend to decrease the NF-κB signal in aged segments, blockade of the HRs induced more remarkable changes. In our experimental settings, the level of NF-κB activation in the HR blockers-treated aged segments was not significantly different from those of control aged segments (p=0.07); however, blockade of HRs was able to reduce the NF-κB signal in 24-mo segments to a level not significantly different from those observed in control 9-mo segments. These findings are represented in the graph on Figure 5. Cumulatively, these data demonstrate that, in adult mesenteric tissues located close to MLVs, NF-κB activation appears to be a mast cell activation-dependent and histamine-dependent process. In the aged mesenteric tissues located close to MLVs, similar to the acute inflammation induced by LPS, the chemical activation of MCs by compound 48/80 was only able to slightly increase the NF-κB activation, because activation levels are already remarkably high under resting conditions. At the same time, we found that in aged mesenteric segments, blockade of HRs not only completely blocked any acute NF-κB activation, but also reduced the aging-associated chronic activation of NF-κB signaling.

### Aging-associated changes in cytokine production in reference to acute peritoneal inflammation

Because the production of cytokines is tightly linked (directly or indirectly) to the activation of NF-κB signaling, we focused our studies on determining potential changes in cytokine profiles in control aged animals as compared to aged animals treated with LPS as well as to adult animals under both control and inflammatory conditions. For cytokine determinations, we analyzed both serum and an extract of proteins from isolated perilymphatic mesenteric tissue segments containing MLVs.

Table 1 presents a summary of all results obtained in the experiments determining cytokine levels in blood and mesenteric tissues obtained from 9-mo as well as 24-mo old rats before and after 24 hours of an acute LPS-induced peritoneal inflammation. Because this study was aimed at investigating mechanisms of aging-associated changes in the body, for presentation purposes we divided the cytokines into three major groups. Group 1 contains the data on cytokines that demonstrated aging-associated differences under control conditions and/or after one day of acute peritoneal inflammation (with direct comparisons between the corresponding pairs of data). Group 2 represents data on cytokine levels unaffected by aging (with direct comparisons between the corresponding pairs of data) but with changes induced by LPS-induced inflammation. Finally, Group 3 consists of data on cytokine levels whose production was not affected by either aging or acute LPS-induced peritoneal inflammation.

**Table 1:** Aging-associated changes in cytokine profiles in blood and mesenteric tissues (including mesenteric lymphatic vessels) in control (CTRL) conditions and after one day of an acute peritoneal inflammation induced by intraperitoneal injection of LPS

Cytokine	Concentration (pg/ml) at various ages / experimental conditions.							
	9 mo				24 mo			
	Blood		Mesentery		Blood		Mesentery	
	CTRL	LPS	CTRL	LPS	CTRL	LPS	CTRL	LPS
	**Group 1. Cytokines with presence of aging-associated differences in control conditions (CTRL) and/or after one day of an acute peritoneal inflammation (LPS)**. **Subgroup 1A: In aged blood/tissues: higher basal concentrations and lower response to LPS**.							
**MIP-1α**	5.2 ± 2.2	88.3 ± 29.9 (*)	6.4 ± 1.3	288.8 ± 82.1 (*)	14.2 ± 5.6	36.7 ± 14.4	13.6 ± 0.1 (#)	133.0 ± 42.2 (#*)
**IL-6**	27.4 ± 17.7	519.9 ± 262.7 (*)	401.7 ± 17.9	462.3 ± 56.5	223.0 ± 56.7 (#)	956.7 ± 372.6 (*)	403.6 ± 62.3	566.9 ± 144.1
**IL-13**	18.9 ± 1.9	41.6 ± 12.7 (*)	32.5 ± 1.9	33.5 ± 2.3	40.0 ± 5.8 (#)	58.2 ± 7.4 (*)	32.7 ± 5.8	37.0 ± 4.1
**IL-17A**	5.9 ± 1.6	40.2 ± 12.1 (*)	38.1 ± 2.9	43.5 ± 9.8	26.6 ± 7.4 (#)	43.4 ± 3.6 (*)	39.7 ± 13.5	38.2 ± 9.0
**MCP-1**	807.2 ± 49.3	6563.7 ± 1267.2 (*)	180.4 ± 4.3	763.4 ± 131.4	1771.6 ± 449.6 (#)	4187.8 ± 982.6 (#*)	179.6 ± 18.3	523.8 ± 110.3
**IP-10**	331.9 ± 50.7	1868.9 ± 96.4 (*)	46.5 ± 2.5	547.8 ± 189.8 (*)	1014.6 ± 248.4 (#)	1687.6 ± 137.0 (*)	54.9 ± 14.7	218.6 ± 81.0
	**Subgroup 1B: In aged blood/tissues: higher basal concentrations and absence of response to LPS**.							
**Eotaxin**	8.8 ± 2.3	20.2 ± 3.9 (*)	26.5 ± 1.3	25.0 ± 2.4	19.6 ± 3.3 (#)	25.5 ± 1.1	23.9 ± 1.8	25.9 ± 2.7
**IL-4**	13.6 ± 4.8	39.9 ± 14.5 (*)	85.6 ± 9.9	78.4 ± 21.1	52.5 ± 10.7 (#)	60.4 ± 27.8	77.6 ± 32.8	68.7 ± 9.7
	**Subgroup 1C: In aged blood/tissues: unchanged basal concentrations and lower response to LPS**.							
**IL-1α**	9.0 ± 6.9	30.1 ± 23.2	13.2 ± 13.2	211.3 ± 60.3 (*)	20.7 ± 10.1	43.4 ± 35.6	25.1 ± 25.1	49.0 ± 19.6 (#)
**KC**	139.1 ± 41.9	3239.4 ± 1335.2 (*)	49.2 ± 49.2	133.3 ± 84.8 (*)	251.3 ± 107.8	1016.1 ± 147.8 (#)	72.7 ± 72.7	62.7 ± 62.7
**IL-1β**	16.0 ± 5.2	109.4 ± 22.4 (*)	61.7 ± 6.7	346.9 ± 63.3 (*)	93.3 ± 59.9	406.7 ± 109.1 (#*)	68.9 ± 11.1	330.8 ± 69.3 (*)
	**Subgroup 1D: In aged blood/tissues: unchanged basal concentrations and higher response to LPS**.							
**TNF-α**	5.7 ± 0.8	11.9 ± 0.6 (*)	10.9 ± 1.9	10.6 ± 1.7	8.2 ± 1.7	15.1 ± 2.9	12.9 ± 2.8 (*)	18.1 ± 4.3 (#)
**VEGF**	78.0 ± 16.4	71.8 ± 5.2	32.8 ± 7.2	71.1 ± 12.8	107.6 ± 13.4	255.8 ± 104.1 (#*)	27.8 ± 13.9	52.6 ± 11.9
**RANTES**	772.7 ± 288.4	554.8 ± 91.9	219.3 ± 116.0	250.9 ± 21.3	2149.9 ± 1154.4	3491.7 ± 1715.0 (#)	1830.7 ± 340.7	1478.7 ± 840.1
	**Subgroup 1E: In aged blood/tissues: higher basal concentrations and higher response to LPS**.							
**IL-12p70**	16.3 ± 5.4	36.4 ± 1.4 (*)	46.2 ± 3.1	47.6 ± 5.2	50.6 ± 21.3 (#)	74.9 ± 25.6 (#)	45.8 ± 9.4	51.8 ± 5.9
**IFNγ**	56.6 ± 10.6	114.9 ± 31.7 (*)	190.8 ± 12.4	245.4 ± 24.0 (*)	170.7 ± 31.3 (#)	363.1 ± 98.5 (#*)	235.1 ± 32.6	232.2 ± 36.5
	**Group 2: Cytokines without aging-associated differences but with changes induced by LPS**.							
**IL-5(+)**	81.3 ± 5.5	166.6 ± 10.4 (*)	96.3 ± 12.2	119.5 ± 23.9	134.0 ± 28.1	194.7 ± 31.0	98.8 ± 28.6	128.2± 41.9
**Leptin (+)**	31022.4 ± 3639.0	55685.3 ± 6559.0 (*)	27749.8 ± 9654.0	46949.6 ± 13324.0	27018.7 ± 5586.0	43284.9 ± 13251.0	15503.7 ± 13366.0	17053.5 ± 9723.0
**IL-18(+)**	154.0 ± 82.1	418.0 ± 105.8 (*)	614.7 ± 90.3	2585.2 ± 346.4 (*)	170.4 ± 47.6	704.2 ± 325.3 (*)	1246.8 ± 369.7	3012.6 ± 945.2 (*)
**Fractalkine**	48.1 ± 5.9	154.9 ± 24.7 (*)	47.9 ± 6.8	155.1 ± 46.0 (*)	51.3 ± 9.8	138.2 ± 41.6 (*)	45.9 ± 18.8	111.3 ± 52.4
**IL-10**	35.7 ± 6.2	612.9 ± 224.9 (*)	34.5 ± 4.8	54.2 ± 15.3	148.9 ± 62.7	1203.0 ± 470.5 (*)	40.4 ± 16.8	44.3 ± 12.6
	**Group 3: Cytokines without aging-associated and/or LPS-induced differences**.							
**G-CSF**	0.4 ± 0.2	7.3 ± 7.0	16.9 ± 0.8	24.1 ± 4.0	0 ± 0	6.9 ± 4.4	21.5 ± 3.1	22.8 ± 4.6
**MIP-2**	76.6 ± 27.4	127.4 ± 82.9	323.8 ± 53.7	438.6 ± 80.5	120.5 ± 26.4	93.1 ± 63.7	354.0 ± 64.9	410.9 ± 65.8
**GM-CSF**	63.0 ± 23.2	70.3 ± 43.1	61.8 ± 51.7	109.0 ± 66.8	81.3 ± 28.2	63.0 ± 63.0	89.3 ± 89.3	72.7 ± 54.6
**IL-2**	61.1 ± 40.3	10.1 ± 4.9	40.4 ± 9.6	57.9 ± 17.4	28.7 ± 16.1	264.3 ± 218.9	52.9 ± 26.6	50.9 ± 19.1
**EGF**	0.3 ± 0.2	0.9 ± 0.8	0.3 ± 0.3	0.9 ± 0.7	0.3 ± 0.2	0.2 ± 0.2	0.6 ± 0.6	0.4 ± 0.4
**LIX**	600.2 ± 131.9	986.3 ± 332.	118.5 ± 10.6	256.8 ± 122.2	679.5 ± 161.6	1155.5 ± 90.2	123.6 ± 34.1	194.5 ± 31.4

Values are means ± SEM; 9 mo – samples from 9 mo-old animals (n=6 for control and n=5 LPS-treated rats), 24 mo – samples from 24 mo-old animals (n=6 for control and n=5 LPS-treated rats). + indicates an absence of aging-associated changes based on paired comparisons but still the presence of certain effects of aging (please see Results section). # indicates a significant difference (p < 0.05, one-way ANOVA) between adult and aged samples in similar types of samples (blood or mesenteric tissue) with similar treatment. * indicates a significant difference (p < 0.05, one-way ANOVA) between the control samples and samples after LPS-induced inflammation in similar types of samples (blood or mesenteric tissue) within each age group.

We found that production of 16 cytokines out of 27 studied was directly affected by aging. Subgroup 1A in Table 1 includes cytokines for which we observed a higher basal concentrations and lower response to LPS in aged animals. We found that in 24-mo old animals, the LPS-induced increase in the levels of MIP-1ɑ this cytokine was ~2.6-fold in blood and ~9.8-fold in mesenteric tissues, while in 9-mo rats the corresponding increases were ~17.0-fold and ~45.1-fold. Production of IL-6, IL-13, IL-17A and IP-10 was changed by aging generally in a similar manner. For example, as a result of such change, LPS-induced inflammation caused a ~19.0-fold, ~2.2-fold, ~6.8-fold and ~5.6-fold increase in these cytokines in blood, respectively, while in aged animals these increases were only ~4.3-fold, ~1.5-fold, ~1.6-fold and ~1.7-fold, respectively. Measurements of eotaxin and IL-4 levels (subgroup 1B in Table 1) indicated higher basal concentrations and absence of a response to LPS in aged animals that was observed in 9-mo animals (~2.3-fold and ~3.0-fold increase after 24 hours of LPS-induced inflammation in blood, correspondingly). Measurements of IL-1ɑ,KC and IL-1β levels (subgroup 1C in Table 1) demonstrated unchanged basal concentrations and a lower response to LPS in aged animals. Importantly, the level of IL-1ɑwas greatly (~16.0-fold) increased in 9-mo rat mesenteric tissues after 24 hours of LPS-induced inflammation, but remained unchanged in aged rats. Opposite to IL-1ɑ, the levels of KC were increased in aged animals in response to LPS-induced inflammation, but this increase (~4.0-fold) was much smaller than observed in 9-mo old animals (~23.3-fold). Similar patterns of lower response to acute inflammation in aged animals we also observed for IL-1β, which level after 24 hours of LPS-induced inflammation in the blood of 24-mo rats was increased ~4.4-fold, while in 9-mo rats the increase was ~6.8-fold. In tissues, these increases were ~4.4-fold in 24-mo rats versus ~5.6-fold in 9-mo animals. For the next group of cytokines, TNF-ɑ, VEGF and RANTES (subgroup 1D in Table 1), we observed no change in basal concentrations and a higher response to LPS in aged animals. For TNF-ɑ, we observed significantly its greater levels in the mesenteric tissues after 24 hours of LPS-induced inflammation: ~1.7-fold higher in aged rats than in adult rats. For VEGF and RANTES, we observed significantly greater levels in the blood of aged animals after 24 hours of LPS-induced inflammation: ~3.6-fold and ~6.3-fold higher in aged rats than in adult rats, respectively. The last two cytokines, whose levels showed statistically significant differences between aged and adult rats within the pairs of data obtained in similar experimental conditions, were IL-12p70 and IFNγ. These cytokines are segregated to subgroup 1E in Table 1 as cytokines with higher basal concentrations and a higher response to LPS in aged animals. In aged animals, we found higher blood levels of these two cytokines than in 9-mo rats. In control conditions, the levels of these two cytokines in the blood of aged animals were ~3.1-fold and ~3.0-fold more than in adult rats, correspondingly. After 24 hours of LPS-induced inflammation the levels of IL-12p70 and IFNγ in the blood of aged rats also appeared to be larger than in the blood of adult rats: ~2.0-fold and ~3.2-fold.

The next five cytokines (IL-5, leptin, IL-18, fractalkine and IL-10), presented in Table 1 as Group 2, were cytokines displaying no aging-associated differences but with changes induced by LPS. It is important to mention that in cases of IL-5, leptin and IL-18 the statistical analysis of any pairs of data (blood or tissue under control conditions or after LPS administration) did not reveal the presence of aging-associated changes (hence supporting their segregation to group 2). However, in 9-mo animals, the level of IL-5 in blood was significantly increased (~2.0-fold) after 24 hours of LPS-induced inflammation, while in blood of aged animals the difference between the levels of IL-5 in the control group and LPS-affected group was not significant. Likewise, we found similar changes in levels of leptin. In 9-mo animals the level of leptin in blood was significantly increased (~1.7-fold) after 24 hours of LPS-induced inflammation while in blood of aged animals the difference between the levels of leptin in control group and LPS-affected group was not significant. For IL-18 we observed LPS-induced increases of its levels in both age groups, with significantly greater values of this cytokine found in mesenteric tissues in control conditions as well as after development of acute peritoneal inflammation. Noteworthy, in 9-mo animals, the level of IL-18 in mesenteric tissues was significantly increased (~4.2-fold) after 24 hours of LPS-induced inflammation while at the same time a similar increase of IL-18 in aged mesentery was also significant but less (~2.4-fold) than in adult tissues. When measuring levels of fractalkine, we found increases in its levels both in blood and in mesenteric tissues after LPS administration in both age groups. Similar changes, but only for blood samples, were observed in measurements of IL-10. The remaining six cytokines studied (G-CSF, MIP-2, GM-CSF, IL-2, EGF and LIX, Group 3 in Table 1) did not demonstrate any aging-associated or LPS-induced changes in their levels.

## DISCUSSION

### Abolished reactivity of aged MLVs to LPS-induced acute peritoneal inflammation

Reports from the literature uniformly demonstrate that LPS (endotoxin) applied to isolated bovine [42] or rat [36] MLVs induces their relaxation and profoundly inhibits their phasic spontaneous contractions. *In vivo* models of gut [43] or peritoneal [36] inflammation revealed similar types of changes in tone and contractility of MLVs. Overall, our data demonstrated similar responses to acute LPS-induced peritoneal inflammation obtained in 9-mo (i.e. adult rats in this study) and those observed in young ~3-mo rats [36]. Nonetheless, the consequence of events in the initial phases of the response of contracting MLVs to LPS, we believe, depends on its initial route of exposure to MLVs – intraluminal (gut inflammation) versus extraluminal (peritoneal inflammation): alterations of contractility of MLVs after 3 days of gut inflammation (with profound inhibition of pump strength/stroke volume at minimal perfusion rate of 0.5 μl/min) [43] appear to be more severe than those observed after 3 days of peritoneal inflammation (with absence of passive perfusion) [36]. Such differences may likely depend on which cell types makes contact with the pathogen initially. Lymphatic endothelial cells are likely the first cells in mesenteric tissues to be exposed to pathogen in the early phases of gut inflammation (recently reviewed in [44]). In contrast, in the early phases of acute peritoneal inflammation, mast cells, not lymphatic endothelial cells, will contact the pathogen first in mesenteric tissues, and therefore the functional status of these mast cells will be critically important during such pathology [6, 8, 9].

Our studies of contractile function in aged MLVs after 24 hours of acute peritoneal inflammation reveal that in aged MLVs, LPS-induced acute peritoneal inflammation did not induce significant changes in phasic lymphatic contractility or lymphatic tone (Figure 1). We believe that the lack of reactivity of the aged contracting mesenteric lymphatic vessels to acute inflammation imposes serious limitations in their functional reactions to changes in the surrounding tissue microenvironment, such as the appearance of pathogen. In response to acute inflammation, MLVs face greater functional demands in order to respond to the inflammation-related tissue edema [43, 45]. These adaptive reserves are significantly limited in aged MLVs, creating a pre-disposition to earlier development and/or worsening of swelling of peripheral tissues with partial or complete loss of lymph transport function. In encapsulated organs this can induce or worsen pain and corresponding distress. In terms of the impaired barrier function of aged lymphatic vessels for the pathogens demonstrated by us earlier [35], the pre-existing relaxation/inhibition of aged lymphatic vessels eases the dissemination of pathogen in tissues compartments by slowing active lymph flow component and therefore increasing the contact time of pathogen with the lymphatic wall. Other work we have done using cultured lymphatic endothelial cells (LEC) monolayers [46] indicated that many of the cytokines we found elevated in aged blood or mesenteric tissues in this study can increase LEC permeability. At the same time, since active lymph flow will be greatly decelerated, lymph flow-directed delivery of pathogen and trafficking of immune cells/signals towards lymph nodes will also be diminished. Additionally, in cases of maximal severity of acute inflammation, it is highly likely that the transport failure of aged lymphatic vessels may happen much earlier, therefore further decreasing lymph flow and worsening the immune response to pathogen.

Corresponding investigations of the alterations in contractile function of aged lymphatic vessels should be more systematically performed to better characterize the sequence of alterations in lymphatic contractile function, lymph flow and trafficking of immune cells via lymphatic vessels with accurate reference to the origin, nature and initial appearance of pathogen, and severity of inflammation. Finally, the regional heterogeneity of lymphatic contractility, biomechanical properties and underlying molecular regulatory mechanisms, already widely documented over the last decade [47–51], should also be taken into account.

### Diminished activation of aged mast cells during LPS-induced acute inflammation

Previously, we quantitatively confirmed that mast cells are located in close proximity to rat MLVs, with a 4.5-fold greater density near MLVs than in mesentery remote to MLVs [8]. We also determined that the pre-existing activated state of these aged mast cells diminishes their ability to react upon subsequent acute exposure to inflammatory stimuli [8]. More recently, we clearly demonstrated that the inflammatory recruitment of MHC class II positive cells and eosinophils towards MLVs requires activation of mast cells, and that this process is diminished in aged tissues in parallel with the diminished ability of chronically pre-activated aged mast cells to react to acute stimulation [9]. It is important to mention that these earlier studies [8, 9] were performed *ex vivo* using tissue culture techniques [52] that did not include blood flow, neural, humoral or the full complement of immunological components. In current study, in order to extend and strengthen our previous conclusions, we used an *in vivo* model of LPS-induced acute peritoneal inflammation that includes all systemic components. We found (Figure 2) that the *in vivo* response of aged mast cells to LPS is greatly diminished due to pre-existing chronic activation, similar to what we demonstrated in *ex vivo* studies for different inflammation-like stimuli [8, 9]. Utilizing a different experimental technique (flow cytometry-based experiments), we further documented substantial pre-existing basal activation in aged mast cells (Figure 3).

When compared to adult counterparts, the fluorescence signal of mast cell tryptase in live, aged mast cells was significantly diminished in control conditions, indicative of MCT release from them. Importantly, 24 hours of LPS-induced acute peritoneal inflammation in aged animals did not induce a further decrease of the MCT fluorescence signal. We believe this is an indication of significant basal degranulation of the aged mast cells and the concomitant release of MCT (and potentially other mast cell-derived mediators) into the surrounding tissue space. Cumulatively, our new data provide further strong support of the increased basal activation of the aged mesenteric mast cells *in vivo*.

### Diminished activation of NF-κB in aged mesentery during LPS-induced acute inflammation

Mast cells play a major role in the pathogenesis of most inflammatory diseases [53, 54]. The initial reactions of mast cells to various inflammatory disorders are most often triggered through the FcεRI receptor on mast cells, with subsequent release of inflammatory mediators from them, including histamine [55]. This induces activation of mast cell-intracellular NF-κB signaling with subsequent secretion of various cytokines, resulting in the induction of allergy and anaphylaxis [56]. Yet, the direct links between mast cell activation, the release of histamine, and the subsequent activation of NF-κB signaling in the surrounding tissues are not established. In particular, the mechanistic interactions between the release of mediators by mast cells close to MLVs, the activation of NF-κB signaling in these tissues, as well as the effects of aging on these mechanisms, are unknown.

In this study, for the first time, we quantitatively evaluated the aging-associated changes in activated/phosphorylated NF-κB (p65) in perilymphatic mesenteric tissue segments under control conditions and after 24 hours of LPS-induced peritoneal inflammation. Our results clearly show low basal levels of NF-κB activation in adult perilymphatic tissues that is profoundly increased upon acute inflammatory stimuli-tion (Figure 4). Imaging these events demonstrates NF-κB activation in a variety of cell types, not only within but also outside of MLVs. These findings extend our understanding of NF-κB signaling upon LPS exposure from inside cultured lymphatic muscle cells [36] to whole tissues in vivo. Also, for the first time, we demonstrate the profound basal activation of NF-κB and very low levels of additional activation following 24 hours of LPS exposure in aged perilymphatic mesenteric tissues. This highlights the diminished ability of cells in aged MLVs and perilymphatic mesenteric tissues to react acutely to NF-κB-activating stimuli.

### NF-κB activation in mesenteric tissues follows the activation of mast cells and histamine in both adult and aged animals

The patterns of changes in NF-κB signaling after 24 hours of acute inflammation in both adult and aged perilymphatic mesenteric tissues (Figure 4) appears to be similar to changes in the functional status of both adult and aged mast cells studies under similar experimental conditions (Figures 2 and 3). The pre-existing basal activation of mast cells in aged perilymphatic mesenteric tissues under control conditions co-exists with pre-existing basal activation of NF-κB signaling. At the same time, 24 hours of LPS-induced acute peritoneal inflammation, as predicted [8, 9], induced less activation of both mast cells and NF-κB signaling in aged perilymphatic mesenteric tissues. Therefore, combining these findings with literature data, we attempted to answer the following question with these studies – are activation of mast cells and release of histamine (selected here as a major mast cell-derived substance released upon their activation) necessary for the activation of NF-κB signaling in both adult and aged perilymphatic mesenteric tissues? The corresponding results (Figure 5) clearly indicate that in adult perilymphatic mesenteric tissues both mast cell stabilization and the blockade of all types of HRs are able to eliminate the acute inflammation-induced activation of NF-κB signaling. Consequently, we conclude that **mast cells trigger NF-κB-mediated reactions of mesentery to acute inflammation through release of histamine**. In aged perilymphatic mesenteric tissues, both mast cell stabilization and blockade of all types of HRs were also able to abolish the acute inflammation-induced activation of NF-κB signaling (though the basal level of NF-κB activation was elevated and the acute inflammation-induced fraction of the activation of NF-κB signaling was diminished in aged tissues). Moreover, blockade of all types of HRs implemented in aged mesenteric tissue segments was able to reduce the aging-associated chronic inflam-mation-induced NF-κB signaling. Taking into account the likelihood that aging-associated basal activation of NF-κB signaling may be multifactorial and the experimental time interval (5 hours) selected for these experiments could potentially be too short, we conclude that **the aging-associated activation of mast cells maintains chronic histamine-dependent activation of NF-κB signaling in aged perilymphatic mesenteric tissues and limits NF-κB activation in aged mesentery in response to acute inflammation**.

### Aging-associated alterations in cytokine production in reference to acute peritoneal inflammation

Pro-inflammatory cytokines are strong predictors of age-related morbidity and mortality [57]. There is accumulating evidence that the local production of inflammatory cytokines drives pathologies associated with aging. The local (tissue) cytokine milieu is an important component of age-related pathologies where the SASP (senescence-associated secretory phenotype) of damaged or senescent cells disrupts local tissue structure and function [58–60]. Given the fact that the NF-κB is one of the major players in regulation of SASP [61–63], further analysis of circulating levels of inflammatory cytokines compared to their levels in the tissue microenvironment requires continuous research efforts. In this study the analysis of 27 major pro-inflammatory cytokines in blood and perilymphatic tissue samples obtained from pre- and post-inflammatory adult and aged rats revealed complex changes due to the acute peritoneal inflammation and variations in their local tissue versus general body production/distribution. The overwhelming number of variables in these results and well-known multifactorial crosstalk of numerous cytokines involved in the inflammatory response of the body to acute inflammation make the task of understanding the functional importance of the current findings quite challenging and extensive follow-up studies are required.

However, initial analysis of this massive data set led us to several important conclusions. First, at least for 18 out of 27 cytokines (the first 18 cytokines in Table 1), the patterns of their acute peritoneal inflammation-induced changes were affected by aging. These changes in certain cytokines (described in Results and below) matched the correlation between activation of mast cells and activation of NF-κB signaling, as discussed above (high basal activation in resting aged tissues and reduced response to acute inflammatory stimuli), but not in all. With knowledge of the multifactorial effect of activated mast cells themselves, and multidirectional effects of activated NF-κB on cytokine production and local versus systemic distribution of cytokines, a mismatch between the dynamics of activation of mast cells and NF-κB in aged inflamed tissues and production of certain cytokines is not surprising. However, an important fact is that production of all 18 of the aging-affected cytokines has been shown by numerous studies to be controlled by activated NF-κB. Because, as demonstrated for the first time in this study, activation of mast cells and histamine availability is necessary for this NF-κB activation, these cytokines are consequently dependent on mast cell activation and histamine release.

Another interesting finding is that the blood level of the cytokine IL-4, generally accepted to represent a Th2 type response, goes up with LPS stimulation in control adult animals, but remains unchanged in aging animals. This result may be important if the increase in IL-4 is a reflection of a compensatory response to the pro-inflammatory cytokines that is missing with age, thereby resulting in a condition of chronic inflamma-tion. Similar to the observations from blood samples, the failure of LPS to increase IL-4 levels upon LPS stimulation in aged animals is consistent with the notion that there is a failed compensatory response to pro-inflammatory cytokines with age. Likewise, in terms of tissue levels of cytokines that may contribute to a compensatory anti-inflammatory balance, the failure of LPS to generate increases in IL-1ɑ and its weak effect on IL-6 and IL-13 in aged animals supports the inter-pretation that aging results in a loss of compensatory anti-inflammatory responses. At the same time, mast cells have been shown to be important effector cells in Th2-dominant immune responses associated with IgE antibody production [64]. It has been demonstrated that mast cells produce a variety of cytokines that, in part, overlap with the production of cytokines by Th2 cells [64]. IL-13, as one of such cytokines, was initially described as a cytokine that is produced by activated Th2 cells, but its expression by activated mast cells has also been reported [65, 66]. Our data demonstrated that IL-13 level in blood from aged rats was significantly higher compared to adult rats. We believe that such a finding indicates a likely shift to a Th2 response in aging, potentially as a mechanism to control the initial pro-inflammatory response. Taken together, the IL-13-mediated events demonstrate the complexity of effects of the mast cell/histamine/NF-κB axis on regulation of the immune response in adult and aged mesentery.

### Mast cell/histamine/NF-κB axis and lymphatic functions

With the underlying principle that lymph transport via lymphatic vessels [67, 68] is an essential function for all other lymphatic functions, we considered some of our current findings in their broader context. For example, it is important to investigate direct effects of LPS on the contractile function of lymphatic vessels, which appears to be to some degree similar in various species and in different experimental settings ([36, 42] and this study). However, acute inflammatory events *in vivo* are considerably more complex than in *ex vivo* settings. Consequently, the mechanisms of the alterations of lymphatic contractility caused by LPS-induced peritoneal inflammation *in vivo* could be quite different than the mechanisms of the LPS-induced alterations of lymphatic contractility *ex vivo*. Therefore, while still being undoubtedly important, data on mechanisms involved in the effects of LPS on cultured lymphatic muscle cells [36] represent only one part of its direct effects on MLVs (i.e., not linked to interactions with lymphatic endothelial cells), and cannot represent the complex balance of effects of LPS-induced acute peritoneal inflammation brought about by degranulation of mast cells and the action of numerous substances activated by the NF-κB signaling system outside of MLVs. For instance, mast cells can produce, store, and release, upon activation, numerous bio-active and, in particular, vasoactive mediators that continuously influence the surrounding tissues [10, 11, 27–29, 69–74]. In the past, some of these vasoactive mediators have been tested, via external delivery to lymphatic vessels, for their ability to influence lymphatic contractility. In particular, data from the literature suggest that histamine is a potent dose-dependent modulator of lymphatic contractility [30, 75–80]. At higher concentrations, histamine acts synergistically with the lymphatic-relaxing effects of nitric oxide, but, at lower concentrations, histamine acts predominantly anta-gonistically to nitric oxide and stimulates lymphatic contractility [30, 75–85]. However, all of these studies demonstrated the influence of these mediators delivered not from mast cells but after direct delivery to a bath with isolated lymphatic vessels. Until now, only one study [30] linked pharmacologically-induced activation and degranulation of mast cells to subsequent stimulatory changes in lymphatic contractility induced by histamine, without reference to its concentration. Therefore, while it is theoretically assumed that during the development of acute peritoneal inflammation the activated mast cells somehow influence lymph contractility and flow, there are no currently available data directly linking release of mediators from mast cells activated by such inflammation with subsequent alterations of the contractility of neighboring MLVs. In addition, the underlying molecular mechanisms have not yet been discovered, but without a doubt are complex and require careful detailed investigations with reference to cause, initial localization and severity of such inflammation.

Similarly, viewed through a myriad of known vasoactive effects of the NF-κB-controlled cytokines, the task of discovering the molecular mechanisms underlying the effects of LPS-induced peritoneal inflammation of lymphatic contractility is not trivial but achievable through careful discovery-driven research. This study demonstrates that the logical central node of this research endeavor belongs to the mast cell/histamine/NF-κB axis. The aging-induced alterations in the functioning of this axis, demonstrated in this study, co-exist with abolished direct effects of LPS on lymphatic contractility in the elderly population. This shifts the task of investigating the effects of aging-associated alterations of the mast cell/histamine/NF-κB axis on lymphatic contractile function towards translational approaches with reference to their effects not only on the maintenance of fluid homeostasis but also to the ability of lymphatic vessels to support adequate transport for immune cells that enter lymphatic vessels during acute inflammation on their way towards lymph nodes.

### Immune cell trafficking towards/through collecting lymphatic vessels and lymphatic permeability/barrier function

Histamine itself has been historically known for its important roles for immune cell functioning, including multi-directional crosstalk between mast cells and macrophages and chemoattraction of various immune cells [86–90]. At the same time, histamine also increases lymphatic permeability [91–93]. With respect to these roles of histamine, we believe that our previously published and current data emphasize its crucial involvement in inflammatory events in lymphatic vessels and perilymphatic tissues. However, data presented in this manuscript also allow widening of the knowledge of the involvement of the mast cell/histamine/NF-κB axis in the regulation of immune cell trafficking towards/through collecting lymphatic vessels and lymphatic permeability/barrier function during acute inflammation.

Recently, we demonstrated that the functional status of mast cells neighboring MLVs is important for recruitment of MHC class II positive cells and eosinophils towards lymphatic vessels in cases of acute inflammatory stimulation [9]. Aging alters this process thereby decreasing the proper trafficking and activation of these immune cells in aged mesenteric perilymphatic tissues [9]. Our conclusion in the current study is that mast cells trigger the NF-κB-mediated reactions of mesentery to acute inflammation through histamine-involved regulatory mechanism(s). When considering some recent important findings in the field of lymphatic biology, we also assign a central role to the mast cell/histamine/NF-κB axis in lymphatic-related inflammatory events. In particular, we believe that novel data on LPS-induced modulation of neutrophil recruitment and macrophage polarization on lymphatic vessels [36] serve as convincing confirmation of our previous data discussed above [9] (macrophages, including CD11b positive cells, belong to MHC class II positive cells ([94] etc.)). In relation to the crucial role of the mast cell/histamine/NF-κB axis in this process and with consideration of the well-known role of NF-κB in the activation of CD11b positive cells [95], we believe that the smaller number of mast cells available for acute inflammatory activation in elderly individuals will diminish NF-κB-driven CD11b cell activation. Since the CD11b positive cells could be monocytes, macrophages, or even activated neutrophils [96], the compromised mast cell/histamine/NF-κB activation in the elderly alters the innate immune response in aged mesentery.

Another important process controlled by the mast cell/histamine/NF-κB axis appears to be dendritic cell trafficking towards lymph nodes. This process is largely controlled by CCR-7 expression on dendritic cells and CCL21 secretion by the lymphatic endothelium [97–100], which has been demonstrated to be NF-κB/TNFɑ-dependent [101]. Furthermore, it has been demonstrated that PI3K/Akt1 and NF-κB signaling, but not signaling through members of the MAPK family, are important in CCR-7/CCL21-mediated survival and migration of dendritic cells [102]. In the healthy adult body, the mast cell/histamine/NF-κB axis is in fine balance between activated and non-activated states. Such balance allows proper control of dendritic cell function. In aging, the normal balance in the mast cell/histamine/NF-κB axis is altered, so basal NF-κB signaling tends to be in a constitutively active state. As a result, the NF-κB-dependent increased basal reactivity of aged dendritic cells to self-antigens contributes to aging-associated chronic inflammation [103]. In turn, we believe that reactivity of aged dendritic cells to an acute inflammation challenge will be compromised, thereby altering the proper course of events in the immune response to pathogens in the elderly population.

Alterations in immune cells trafficking towards/through collecting lymphatic vessels in the aged body co-exist with the aging-associated decrease in barrier function of lymphatic collectors, which creates a predisposition for easier spread of pathogens towards the perilymphatic tissues [35]. Recently, it has been demonstrated that lymphatic collecting vessel permeability is controlled by CCR7 in an IRF-4-dependent manner [104]. As IRF-4 expression is an NF-κB-dependent process [105], we believe our previously published data on compromised pathogen transport and lymphatic permeability in the aged body [35] clearly underscore the importance of the mast cell/histamine/NF-κB axis as a major contributor to the maintenance of lymphatic permeability by CCR-7/CCL21/IRF-4-dependent dendritic cells [104]. Finally, we believe that our current data on aging-associated and mast cell/histamine/NF-κB axis-dependent changes in cytokine profiles before and after acute peritoneal inflammation provide additional support for the conclusion that control of lymphatic permeability during acute inflammation is a complex multifactorial process [46], which, in our belief, still requires a wide spectrum of in-depth research efforts.

In conclusion, in this study, for the first time we demonstrate that aged MLVs have abolished reactivity to LPS-induced acute peritoneal inflammation, and this change co-exists with diminished activation of aged mast cells inhabiting perilymphatic tissues and decreased activation of NF-κB in aged mesentery. Our results clearly indicate that activated mast cells trigger NF-κB signaling in the mesentery through release of histamine. We conclude that aging-associated basal activation of mesenteric mast cells limits acute inflammatory NF-κB activation in aged perilymphatic mesenteric tissues. Therefore, aging-associated dysfunction of mesenteric mast cells critically affects all NF-κB-mediated reactions of aged mesentery to acute inflammation inclusive but not limited to cytokine production. Proper functioning of the mast cell/histamine/NF-κB axis is essential for the regulation of lymphatic vessel transport and barrier functions as well as for both the interaction and trafficking of immune cells near and within lymphatic collectors. Thus this axis appears to play important roles in alterations in inflammation and immunity associated with aging.

## METHODS

### Animal procedures

For the current studies, we used Fischer-344 (F-344) male rats (obtained from the aged rat colony maintained by the National Institute of Aging at the NIH). The animals represented two age groups: adult (9-month-old [9-mo]) and aged (24-month-old [24-mo]). All animal procedures were reviewed and approved by our Institutional Animal Care and Use Committee and were in accordance with federal and local regulations. The average body weight of the rats used in this study was 438 ± 15 g in 9-mo rats and 418 ± 25 g in 24-mo rats. It should be noted that for the F-344 rat strain, the majority of the weight gain occurs before 7 months of age [106]. Therefore, the weights of both 9- and 24-mo rats are within ranges slightly above 400 g and are not significantly different.

For some experiments we used control rats of both ages to isolate mesenteric tissue segments to be incubated for various periods of time with or without LPS (10 μg/ml). To create acute peritoneal inflammation *in vivo* we used the commonly accepted model of 24 hours of the LPS-induced inflammation [36]. For this purpose, rats of both ages received a single IP injection of LPS (10 μg/kg body weight) or saline (equivalent volume as a sham control).

On the experimental day, all rats were anesthetized with a solution containing a combination of Fentanyl/Droperidol (0.3 mL/kg IM) and Diazepam (2.5 mg/kg IM). Subsequently, the chest was opened (which is a terminal procedure) and blood was collected immediately *post mortem* from the heart for determination of cytokine levels. Immediately after blood collection, a midline abdominal incision was made and the whole gut was removed by cutting at the root of the mesentery after clamping it to avoid excessive bleeding. Mesentery of the small intestine (without gut), containing MLVs, was dissected and used for experiments.

### Analysis of lymphatic contractility

Once exteriorized, the mesenteric segments containing MLVs were transferred to standard 35-mm petri dishes (one segment/dish) completely filled with ~38°C Dulbecco's modified Eagle's medium/F12 (DMEM/F12) solution (Invitrogen Corp., Carlsbad, CA, USA, cat.# 11039) supplemented with antibiotic mixture (Invitrogen Corp., Carlsbad, CA, USA, cat.# 15140) to achieve a concentration of 100 IU/ml of penicillin and 100 μg/ml of streptomycin per ml of DMEM/F12. We have demonstrated that lymphatic contractility is not affected by overnight incubation in this solution [107–109]. Care was taken to ensure that the mesenteric segment was completely submerged under the medium in the dish. The mesenteric segments of both ages were randomly divided into two groups – sham control and LPS-treated (medium supplemented with LPS at 10 μg/ml). For each experimental day we used both control and LPS-treated mesenteric segments obtained from the same animal.

After 24 hours of incubation with or without LPS, the sections of the mesentery were positioned and secured under a stereo microscope to allow isolation of segments of MLVs for functional studies. Isolated MLVs were transferred to an isolated vessel chamber (modified Living Systems Instrumentation Inc. single vessel chamber model CH/1, St. Albans City, VT, USA) filled with pre-warmed 38°C DMEM/F12 (pH 7.36). The isolated MLV segments were cannulated and tied onto two carefully matched glass pipettes (100-110 μm). Great care was used to prepare and select pairs of resistance-matched pipettes for these experiments, as described in our previous studies [5, 48, 110, 111]. The inflow and outflow pipettes were connected to independently adjustable pressure reservoirs filled with DMEM/F12. Care was also taken to ensure that there were no air bubbles in the tubing or the pipettes. Once the vessels were cannulated, a slight positive transmural pressure (3 cm H_2_O) was applied to detect leaks and to ensure that the vessels were undamaged and untwisted. The vessels were set to their approximate *in situ* length and positioned just above the glass coverslip comprising the chamber bottom. The chamber was transferred to the stage of a microscope. The vessels were set to an equilibration transmural pressure of 3 cm H_2_O at 38°C for 15-20 minutes. Once tone and spontaneous contractions were observed, the vessels were allowed to equilibrate at 3 cm H_2_O for another 30 minutes prior to beginning the experiment. During all experiments, MLV segments were constantly superfused with pre-warmed 38ºC DMEM/F12, and the isolated vessel chamber was constantly warmed to maintain a temperature of 38±0.1°C in the DMEM/F12 sur-rounding the vessel. A CCD video camera, monitor, Windows-operated computer supplied by National Instruments data acquisition card NI PCI-1410 and DVD/HDD recorder were used to observe and record the lymphatic segments and to track their diameter continuously in all experiments.

In every experiment, we evaluated the contractile responses of MLV segments at transmural pressures of 1, 3 and 5 cm H_2_O for 5 minutes, the common pressure range used to evaluate contractile function of mesenteric lymphatic vessels [5, 48, 110, 112]. For the sham control, measurements of lymphatic contractile activity were performed with superfusion of DMEM/F12; for the LPS-treated group, superfused DMEM/F12 was supplemented with LPS (10 μg/ml). At the end of each experiment, the passive (relaxed) diameter was measured at each pressure after the vessels were exposed to a nominally calcium-free, EDTA-supplemented physiological saline solution (PSS) (in mM: 145.00 NaCl, 4.7 KCl, 1.17 MgSO_4_, 1.2 NaH_2_PO_4_, 5.0 dextrose, 2,0 sodium pyruvate, 3.0 EDTA, 3.0 MOPS) for 15 minutes.

### Data analysis and statistics for isolated lymphatic vessel experiments

Lymphatic diameters were tracked continuously during experiments using “Vessel Track” software developed previously [113]. We used cardiac pump analogies to define systole and diastole in reference to the lymphatic contractile cycle [48, 114], and the end-diastolic and end-systolic points in the diameter tracings were recorded for each 5-minute interval for a transmural pressure of 5 cm H_2_O and for imposed flow gradients of 1, 3 and 5 cm H_2_O. From the lymphatic end-diastolic and end-systolic diameters (EDD and ESD), the following lymph pump parameters were calculated: lymphatic tone index (the difference between the passive lymphatic diameter in Ca^++^-free PSS and end-diastolic diameter, expressed as a percentage of the passive lymphatic diameter in Ca^++^-free PSS), contraction amplitude (the difference between the diastolic and systolic diameters), contraction frequency, ejection fraction (EF, the fraction of end-diastolic volume ejected during the single lymphatic contraction, calculated using the formula EF=(EDD^2^-ESD^2^)/EDD^2^) [114], and fractional pump flow (FPF, an index of lymph pump flow, calculated as the ejection fraction times the contraction frequency). To compare the changes in diameter during the lymphatic contractile cycle, the diastolic and systolic diameters were normalized to the passive lymphatic diameters in Ca^++^-free PSS at the corresponding transmural pressure because of the anatomical variations between lymphatic vessels. Statistical differences were determined by ANOVA, regression analysis and Students t-test (JMP software version 9.0.2. for Windows, SAS Institute Inc., Cary, NC, USA) and considered significant at p < 0.05. (A similar approach for statistical analysis was used in other groups of experiments described below in which quantification of parameters was possible.) Only one vessel segment was used from one animal for the control and one for the LPS-treated group. We used data from 6 animals of each age group for functional analysis of MLV contractility.

### Ruthenium Red staining of mast cells in isolated mesenteric tissue segments

In order to evaluate the aging-associated changes in the functional status of mast cells located near MLVs, we performed studies of mast cell activation using Ruthenium Red, the commonly accepted stain for degranulated mast cells, as we previously described [8]. Ruthenium Red is a cationic dye that is able to enter degranulated cells only and thus has been used in the past to measure mast cell activation in a quantitative manner [115–119].

The exteriorized gut with mesentery was rinsed in warm (38ºC) standard PSS (in mM: 145.0 NaCl, 4.7 KCl, 2.0 CaCl2, 1.2 MgSO4, 1.2 NaH2PO4, 5.0 dextrose, 2.0 sodium pyruvate, 0.02 EDTA, 3.0 MOPS) with pH adjusted to 7.36. At least 2 segments of mesentery (without gut) from each animal (both 9- and 24-mo) were cut and placed into specially designed tissue chambers [52]. These custom designed chambers were developed to be used to treat equal-sized segments of mesentery in fixed position over time and to allow imaging of tissue structures during several subsequent treatments [8]. The exteriorized mesenteric segments of both ages were separated into two groups – sham control (PSS supplemented) and LPS-treated (10 μg/ml) – and kept in those conditions overnight at 38ºC. For each experimental day, we used both control and LPS-treated mesenteric segments obtained from the same animal.

After treatment, chambers containing live mesenteric segments were washed with warm PSS three times for 5 minutes each and 0.00125% Ruthenium Red (Sigma Aldrich, St. Louis, MO, USA, catalog # R2751) in PSS was added to all chambers. Tissue segments were incubated in Ruthenium Red for 30 minutes at 38°C, then washed with warm PSS three times for 5 minutes each wash and imaged using an Olympus CKX41 fluorescence microscope using bright field mode. Multiple images of each mesenteric segment were taken with some overlap in order to analyze mast cell activation over the entire segment, zone-by-zone.

### Data analysis for mast cell activation experiments

Captured sets of images were analyzed using a Windows-operating computer. Any pink- or red-stained cells were considered to be activated. Such activated cells were manually counted for each image using a regions of interest (ROI) that was purposely selected (using NIH Image J software) to be small enough to move over all zones of mesentery that were clear of adipose tissue (imaging is not possible when adipose tissue is present due to the excessive thickness of the mesentery). All ROIs were of the same size and all mast cells visible within ROIs were counted, being careful not to count more than once those zones which depicted overlap between subsequent images. The numbers of activated mast cells in all ROIs within any one particular image of a given tissue segment were averaged, and then the average numbers obtained from all images of that tissue segment were grouped and averaged again. When we were able to take more than two mesenteric segments from each animal for study, we averaged the numbers obtained in similarly treated (sham or LPS treatment) segments from that animal. For mast cell activation experiments we used data obtained from 4 animals of each age group.

### Flow cytometric analysis of mast cells in isolated mesenteric tissue segments

In order to further assess the functional status of mast cells located near MLVs, we also utilized a different experimental technique - flow cytometric analysis. In this set of experiments, we used the mesenteric tissue segments obtained from animals of both ages in which we induced acute peritoneal inflammation using a single IP injection of LPS (10 μg/kg body weight) or vehicle for the sham control. After 24 hours, we isolated (as described above) the segments of mesentery containing MLVs and mast cells located nearby. After isolation, these segments were incubated with collagenase, Type I, (Worthington Biochemical Corp., Lakewood, NJ, USA, catalog # M3A14008B) (500 units/ml in phosphate buffered saline (PBS, catalog # 6505; EMD Chemicals, Gibbstown, NJ)) for 30 min at 37^°^ C, while slowly rotating samples using an Isotemp Hybridization Incubator (Thermo Fisher Scientific, Waltham, MA, USA, catalog # 13-247-10Q). We filtered the tissue suspension using a 40-micrometer nylon cell filter in order to eliminate large, undigested tissue fragments. Filtered cells were centrifuged for 5 min at 500 x g using an Eppendorf 5804 R centrifuge (Thermo Fisher Scientific, Waltham, MA, USA, catalog # 05-413-109). The pellet was then washed and re-suspended in PBS. Cells were permeabilized using Intracellular Fixation/Permeabilization Kit (eBioscience, Inc., Sand Diego, CA, USA, catalog # 88-8824-00). Flow cytometric analysis was performed as described before [120]. Briefly, cells were stained for mast cell antigens using the following antibodies: rat PerCP/Cy5.5-conjugated anti-c-kit (BioLegend, San Diego, CA, USA, catalog #105823, dilution 1:100) and mouse anti-mast cell tryptase (Abcam, Cambridge, MA, USA, catalog # ab2378, dilution 1:10). LIVE/DEAD® Fixable Aqua Dead Cell Stain (Molecular Probes, Thermo Fisher Scientific Inc., Waltham, MA, USA, catalog # L34957, dilution 1:200) was included to differentiate the live and dead cell populations. The second step for staining of mast cell tryptase was performed using a goat anti mouse IgG antibody conjugated with FITC (Abcam, Cambridge, MA, USA, catalog # 6785, dilution 1:1000). Data were acquired on a BD FACS Canto II flow cytometer (Franklin Lakes, NJ, USA) and the analysis was performed using FlowJo Software (Tree Star Incorporated, Ashland, OR, USA).

### Data analysis for flow cytometric analysis of mast cells

In order to identify mast cells, we adopted a gating strategy in which we first identified singlet cells based on the Forward Scatter-Area against Height; secondly we identified live cells as the Aqua negative population; and finally we identified the mast cells as positive for both mast cell tryptase and c-kit. The total number of these double (mast cell tryptase/c-kit) positive cells was normalized to the total number of live cells determined in each sample. In addition, in these mast cell tryptase/c-kit positive cells, we determined the mean fluorescence intensity (MFI) of both mast cell tryptase and c-kit signals. During this analysis, we considered the loss of tryptase by mast cells (i.e., a decrease in MFI of the tryptase signal) as an indicator of their activation and degranulation [37–39]. For these experiments we collected data from 3 control and 3 LPS-treated rats of each age.

### Immunohistochemical labeling of phospho-NF-κB p65 in isolated mesenteric tissue segments

In order to evaluate the status of activation of NF-κB signaling in adult and aged tissues, in resting conditions and upon development of the LPS-induced acute peritoneal inflammation, and to establish its potential dependence on activation of mast cells and histamine, we performed immunohistochemical labeling of phospho-rylated NF-κB (p65) as a widely used method to evaluate the activation of this signaling pathway [40, 41].

To determine NF-κB activation under conditions of LPS-induced acute peritoneal inflammation the exteriorized mesenteric segments were obtained from animals of both ages, as described above for mast cell activation experiments. To establish links between mast cell activation, histamine and NF-κB activation, the freshly exteriorized mesenteric segments from animals of both ages were separated into 4 groups – (1) sham control; (2) treated with compound 48/80 (10 μg/ml in PSS), an activator of mast cells [8, 121] (Sigma Aldrich, St. Louis, MO, USA, catalog # C2313); (3) treated with compound 48/80 plus cromolyn sodium (25μM in PSS) (Sigma Aldrich, St. Louis, MO, USA, catalog # C-0399). Cromolyn has previously been reported to block mast cell activation and to inhibit histamine secretion from rodent peritoneal mast cells [9, 122, 123]; (4) treated with compound 48/80 plus a mixture of histamine receptor (HR) blockers added to PSS in order to block all 4 types of HRs and to prevent the potential effect of histamine on NF-κB activation. We added a mixture of the following HR blockers: pyrilamine maleate (1 μM [30]) (Sigma Aldrich, St. Louis, MO, USA, catalog # P5514); cimetidine (100 μM [124]) (Sigma Aldrich, St. Louis, MO, USA, catalog # C4522) and thioperamide maleate (1 μM [125]) (Sigma Aldrich, St. Louis, MO, USA, catalog # T123). The mesenteric segments were treated as follows: (1) sham control for 5 hours; (2) sham treatment for 2 hours followed by compound 48/80 for another 3 hours, to induce activation of mast cells; (3) pre-treated with cromolyn sodium for 2 hours, in order to block mast cell activation and to inhibit histamine secretion from rodent peritoneal mast cells, then treated with compound 48/80 plus cromolyn for another 3 hours; (4) pre-treated with the mixture of HR blockers for 2 hours, in order to block all 4 types of HRs, then treated with compound 48/80 plus HR blockers for another 3 hours at 38ºC. For each experimental day we used control, 48/80-treated, 48/80 + cromolyn-treated and 48/80 + HR blockers-treated mesenteric segments obtained from the same animal.

After treatment, all segments were fixed with 4% paraformaldehyde for 30 min at room temperature. All fixed tissues were then washed 3 times with 0.3% Triton X-100 (Sigma Aldrich, St. Louis, MO, USA, catalog # T8787) in PBS for 15 minutes each. Next, segments of mesentery were placed in a 24-well plate and blocked for 1 hour at room temperature with 5% goat serum (Jackson ImmunoResearch, West Grove, PA, USA, catalog # 005-000-121) in 0.1% Triton X-100 in PBS. Incubation with rabbit anti-phospho NF-κB p65 antibody (Cell Signaling, Boston, MA, catalog # 3033, dilution 1:100) was carried out in 0.5% goat serum in 0.1% Triton X-100 overnight at 4°C, followed by washing three times with 0.1% Triton X-100 for 20 minutes each and incubation with anti-rabbit Alexa Fluor 647-conjugated secondary antibody (Invitrogen, Carlsbad, CA Catalog # A21245, dilution 1:200) for 1 hour at room temperature. Finally, after washing all segments 3 times for 20 minutes each in 0.1% Triton X-100 PBS, segments of mesentery were placed on a slide, covered with anti-fade solution (Life Technologies, Carlsbad, CA, catalog # P36934) and imaged using an Olympus DP72 fluorescence camera and an Olympus CKX41 fluorescence microscope. Multiple images of each mesenteric segment were taken with a certain degree of overlap in order to analyze NF-κB activation over the whole segment zone-by-zone.

### Data analysis for NF-κB activation experiments

Captured sets of images were analyzed using a Windows-operating computer. The degree of NF-κB phosphorylation was assessed by measuring pixel intensity of the phospho-NF-κB p65 red signal, using NIH Image J software. The mean pixel intensity was measured in multiple ROIs, which were purposely selected to be 100 μm^2^. The mean pixel intensity in all ROIs within any one particular image of a given tissue segment was averaged (being careful not to include more than once those zones that depicted overlap between subsequent images from one mesenteric segment), and then the average numbers obtained from all images of that tissue segment were grouped and averaged again. To assess NF-κB activation, before and after LPS treatment, we used data obtained from 4 animals of each age group. Likewise, segments from 4 animals of each age group were used for experiments to determine the linkage between mast cell activation, histamine and NF-κB activation.

### Determination of cytokine levels

We measured the levels of 27 major cytokines following induction of acute peritoneal inflammation via a single IP injection of LPS (10 μg/kg body weight) or vehicle for a sham control, using a Multiplex cytokine assay system (EMD Millipore, Billerica, MA, USA, kit #RECMAG65K27PMX) for analyzing mesenteric tissue segments and blood samples obtained from animals of both ages. The mesenteric tissue samples provided information about the status of cytokine production locally, while the cytokine levels in serum reflected the systemic distribution of cytokines. The measurements of certain cytokines in tissues, even after having removed blood from the tissue, were higher than the levels of cytokines measured in blood, which may be indicative to an increased production of these cytokines locally in perilymphatic mesenteric tissues. However, the absolute measured values of tissue-produced cytokines are not as accurate as the levels that may exist *in vivo* due to the diminished volume of blood in harvested tissue segments (as a result of collection of blood samples and loss of blood from these tissues during dissection). At the same time, we did not evaluate the differences in the levels of cytokines between blood and tissue samples directly.

The selected kit allowed us to determine levels of the following cytokines: epidermal growth factor (EGF), eotaxin/CCL11, fractalkine; granulocyte-colony stimulating factor (G-CSF), granulocyte-macrophage colony-stimulating factor (GM-CSF), keratinocyte chemoattractant (KC) [also named Chemokine (C-X-C motif) ligand 1 (i.e. CXCL1)], interferon gamma (IFNγ); interleukin-1alpha (IL-1ɑ); interleukin-1beta (IL-1β); interleukin 2 (IL-2); interleukin-4 (IL-4); interleukin 5 (IL-5); interleukin 6 (IL-6); interleukin-10 (IL-10); interleukin-12 p70 (IL-12p70); interleukin-13 (IL-13); interleukin-17A (IL-17A); interleukin 18 (IL-18); interferon gamma-induced protein 10 (IP-10); leptin; chemokine (C-X-C motif) ligand 5 (LIX); monocyte chemotactic protein-1 (MCP-1); macrophage inflammatory protein-1alpha (MIP-1ɑ); macrophage inflammatory protein-2 (MIP-2); RANTES (chemokine (C-C motif) ligand 5, also named as CCL5); tumor necrosis factor alpha (TNF-ɑ); and vascular endothelial growth factor (VEGF).

### Blood sample processing

At 24 hours following IP injection of LPS (or vehicle), blood was collected from the left ventricle of the heart using a syringe. Blood was transferred to 50-ml plastic tubes and left to clot at room temperature for 30 min in order to collect the serum. Serum was harvested by centrifugation at 2000 rpm (centrifuge Jouan CR4.12, DJB Labcare Limited, Newport Pagnell, UK) and stored at −80°C until analyzed. For analysis, aliquots of serum (25 μl) were loaded into each well of the Multiplex plate, and cytokine analysis was carried out by following the manufacturer's instructions.

### Mesenteric tissue sample processing

After collection of blood, segments of mesentery containing MLVs were collected (four pooled segments with total tissue weight 0.60±0.05 g for each sample). Proteins were isolated from these segments using 200 μl of PBS containing MgCl_2_ (1 mM), Triton X-100 (0.1%); and protease inhibitor cocktail (Sigma Aldrich, St. Louis, MO, USA, catalog # P8340) diluted 1:100. The tissue segments were sonicated for 5 min and then 50 μg of stainless beads 0.5 mm diameter (Next Advance Inc., Averill Park, NY, USA, catalog # SSB05) were added to each sample. Samples were then loaded into a Bullet Blender Tissue Homogenizer (Next Advance, Inc., Averill Park, NY, USA) and vortexed for 5 min at speed level 8. Next, the samples were placed on ice for 1 min. The sonication and centrifuge steps were repeated 2 or 3 times until the tissue was clearly homogenized. Subsequently, samples were incubated for 15 min on ice and then centrifuged at 13000 rpm using an AccuSpin Micro 17 centrifuge (Thermo Fisher Scientific, Waltham, MA, USA, catalog # 05-413-109) for 5 min. A lipid-like top layer was visible after centrifugation (fat tissue) and was removed. The remaining supernatant was harvested (with beads and pellet discarded) and centrifuged again for 2 min at 13000 rpm to remove debris. Protein estimation was performed using a Pierce BCA Protein Assay Kit (Thermo Fisher Scientific Inc., Waltham, MA, USA, catalog # 23225). Isolated proteins were stored at −80°C until analyzed. Aliquots of 30 μg of total protein were loaded into wells of the Multiplex plate for analysis, following the manufacturer's instructions.

### Data analysis for determination of cytokine levels

Wells in Multiplex plates were loaded with serum or tissue extracts in triplicate, with the exception of a few control serum samples (randomly selected from control adult rats) that were loaded in duplicate. All triplicate (or duplicate) readings of samples were averaged and a single average was used for subsequent statistical analysis. Data were collected from 6 control and 5 LPS-treated rats of each age.
